# Editorial: Community series in development and harmonization of assays and models to assess immunogenicity and correlates of protection of vaccines against pathogens causing respiratory infections, volume 2

**DOI:** 10.3389/fimmu.2025.1670957

**Published:** 2025-09-11

**Authors:** Geert Leroux-Roels, Donata Medaglini

**Affiliations:** ^1^ Ghent University, Ghent, Belgium; ^2^ University of Siena, Siena, Italy

**Keywords:** editorial, respiratory pathogen, assays harmonization, correlate of protection, immunogenicity

The development and harmonization of assays and models to evaluate vaccine immunogenicity and define correlates of protection remain critical challenges in the field of vaccinology, particularly for pathogens causing respiratory infections. This second volume of the *Community Series in Development and Harmonization of Assays and Models to Assess Immunogenicity and Correlates of Protection of Vaccines Against Pathogens Causing Respiratory Infections* showcases recent efforts to address these challenges through innovative scientific and regulatory approaches. The contributions presented in this edition explore advanced *in vitro* models, systems immunology, and biomarker discovery, as well as offer regulatory perspectives and novel vaccine strategies—all aimed at enhancing our understanding of immune protection and supporting the rational design and evaluation of next-generation respiratory vaccines.

One publication from the Inno4Vac consortium - a public-private partnership addressing scientific bottlenecks in vaccine development (www.inno4vac.eu) - discusses the development cell-based human *in vitro* 3D models simulating mucosal infection to evaluate vaccine efficacy and immune protection. Ekanger et al., representing the respiratory models team, compared three human respiratory epithelium models - a primary human bronchial epithelial cell derived air-liquid interface transwell model, and two airway organoid models from human airway- and lung-derived adults stem cells - to study infection with influenza A virus, antiviral cytokine responses, and serum-mediated inhibition of infection (1). Despite differences in cell source, culture methods, and infection protocols, these models consistently produced similar infection and neutralization results. This study demonstrates that standardization enhances comparability across different complex *in vitro* 3D culture systems.

Two further articles explore immune responses to SARS-CoV-2 mRNA vaccination and their association with inflammatory biomarkers. In one, Leno-Duran et al. analyzed 92 serum biomarkers in 147 healthcare workers post-BNT162b2 vaccination, uncovering increases in biomarkers related to T cell function (ADA, CD8α, IL-17C, and CCL25) and decreases in markers associated with inflammation and cancer (uPA, IL-18R1, EN-RAGE, CASP-8, MCP-2, TNFβ, CD5, and CXCL10) five weeks after vaccination. Higher MCP-2 levels were associated with higher mean anti-S antibody titers, while HGF and CD6 levels showed negative associations with antibody titers (2).

In another, Lucchesi et al. performed transcriptomic analysis on peripheral blood mononuclear cells from 20 hemodialysis patients and 9 healthy controls before and 7 days after each of two doses of mRNA BNT162b2 vaccine, given three weeks apart (3). Significant gene expression differences related to B cell abundance and regulation, CD4 T cell proliferation, and inflammation pathways were observed at baseline and day 7 between low-responder hemodialysis patients and healthy controls, while high- responder hemodialysis patients displayed an intermediate expression profile for these genes.

Although these studies did not directly focus on assay development or correlates of protection, they highlight the importance of identifying predictive biomarkers and understanding dysregulated pathways in vaccine responses across diverse populations.

Additionally, Buoninfante and Cavaleri provide a regulatory perspective on the importance of eliciting T cell-mediated immunity (CMI) in vaccination, emphasizing the need for standardized methods to measure CMI responses for regulatory submissions (4). Despite assay complexity and variability, the authors advocate for the systematic consideration of T cell responses in vaccine development, especially when CMI is considered to play a role in protection. Advances in technology and ongoing efforts to harmonize assay protocols (5–7) are expected to enable reliable, reproducible measurements of T cell responses to better inform vaccine efficacy, provided the data are validated in clinical studies.

Finally, Victor Cnossen and colleagues introduce the EU-funded FLUniversal consortium, aiming to develop a universal, intranasal live-attenuated influenza vaccine – DeltaFLU- that protects against all influenza strains (8). Preclinical studies and clinical trials, including a controlled human infection model (CHIM), will evaluate safety, immunogenicity and protective efficacy. Comprehensive analyses of blood and mucosal immune responses are anticipated to uncover novel correlates of protection (CoPs).

Together, the studies featured in this Research Topic underscore the importance of integrating multidisciplinary approaches—from advanced *in vitro* models and biomarker analysis to regulatory science and novel vaccine platforms—to better assess immunogenicity and define meaningful correlates of protection. As respiratory pathogens continue to pose significant public health threats, collaborative efforts such as those presented here are essential to drive innovation, standardization, and translational impact in vaccine development.
